# Interacting Chemokine Signals Regulate Dendritic Cells in Acute Brain Injury

**DOI:** 10.1371/journal.pone.0104754

**Published:** 2014-08-25

**Authors:** Charlotte Israelsson, Annika Kylberg, Henrik Bengtsson, Lars Hillered, Ted Ebendal

**Affiliations:** 1 Department of Neuroscience, Developmental Neuroscience, Biomedical Center, Uppsala University, Uppsala, Sweden; 2 Department of Neuroscience, Neurosurgery, Uppsala University, Uppsala, Sweden; Massachusetts General Hospital and Harvard Medical School, United States of America

## Abstract

Brain trauma is known to activate inflammatory cells via various chemokine signals although their interactions remain to be characterized. Mice deficient in *Ccl3*, *Ccr2* or *Cxcl10* were compared with wildtype mice after controlled cortical impact injury. Expression of *Ccl3* in wildtypes was rapidly upregulated in resident, regularly spaced reactive microglia. *Ccl3*-deficiency enhanced endothelial expression of platelet selectin and invasion of peripheral inflammatory cells. Appearance of *Ccr2* transcripts, encoding the Ccl2 receptor, reflected invasion of lysozyme 2-expressing phagocytes and classical antigen-presenting dendritic cells expressing major histocompatibility complex class II. *Ccr2* also directed clustered plasmacytoid dendritic cells positive for the T-cell attracting chemokine *Cxcl10*. A reduction in *Ccr2* and dendritic cells was found in injured wildtype cortex after cyclophosphamide treatment resembling effects of *Ccr2*-deficiency. The findings demonstrate the feasibility to control inflammation in the injured brain by regulating chemokine-dependent pathways.

## Introduction

Traumatic brain injury (TBI) in mice results in distinct upregulation of hundreds of gene transcripts including many linked to inflammation [1], [2]. The inflammatory cascade activated by TBI lacks specific pharmacological treatments and is elicited by mechanisms including reactive oxygen species (ROS), hemorrhage and release of nucleotides sensed by upregulated pyrimidinergic receptors such as P2ry6 [1]. This leads to activation of resident microglia offering an instantaneous response [3]. In addition, invading classical (cDCs) and plasmacytoid dendritic cells (pDCs) [4] become engaged in the inflamed central nervous system. The interactions and signals among these inflammatory cells are not fully understood but one possibility includes a balance among chemokine networks.

Chemokines and chemokine receptors are known to orchestrate inflammatory responses via chemotaxis in injured tissues [5], [6]. We have previously described the appearance of clustered inflammatory cells expressing chemokine *Cxcl10* scattered in grey and white matter of injured wildtype (wt) brains using a controlled cortical impact (CCI) injury model [1], [2], [7], [8]. At the molecular level, strong transcriptional activation after traumatic brain injury can also be seen in chemokine pathways including *Ccl3* (with cognate receptors *Ccr1* and *Ccr5*) and *Ccr2* (with strongly upregulated ligands *Ccl2* and *Ccl12*) as listed in Table 1. Although this suggests postinjury chemokine functions in brain tissue the interactions among different chemokines, cellular sources of signals and targets for signaling remain unknown. In order to establish a possible interplay among chemokine pathways, we studied knock-out *Ccl3−/−*, *Ccr2−/−* and *Cxcl10*−/− mice subjected to TBI. Based on the findings, a model for hierarchical chemokine activities in the injured cerebral cortex is currently presented.

**Table 1:** Chemokine ligands and receptors relevant for this report.

Chemokine	Alternate name	Receptor
***Ccl3***, chemokine (C-C motif) ligand 3	MIP-1α/macrophage inflammatory protein 1 alpha	*Ccr1, Ccr 5*
*Ccl2*, chemokine (C-C motif) ligand 2	MCP-1, monocyte chemotactic protein 1	***Ccr2***
***Cxcl10***, chemokine (C-X-C motif) ligand 10	IP-10, interferon gamma-induced protein 10	*Cxcr3*

In a previous report [2] we demonstrated general similarities among inflammatory responses after TBI and neurodegenerative disorders in mouse models. Our results distinguish the brain injury response in resident microglia cells in the brain parenchyma from the contribution of invading immune cells providing phagocytes, antigen-presenting dendritic cells as well as clustered *Cxcl10*-producing cells. We also investigated posttreatment in injured mice using the immune-suppressing agent cyclophosphamide, clinically administered to patients with severe forms of systemic lupus erythematosus, in order to consider a pharmacological compound for treatment of TBI. Similar to the outcome of TBI in *Ccr2*-deficient mice, cyclophosphamide dampened the increases of dendritic cells and limited markers of antigen presentation in the injured brain. The study design is presented in Table 2.

**Table 2:** Study design.

Aim	Experiments
Outcome of TBI in different chemokine-deficient mouse strains (wildtype versus Ccl3−/−, Ccr2−/− and Cxcl10−/−)	qRT-PCR, microarray expression analysis, *in situ* hybridization, isolectin B4 histochemistry, brain cavity volume measurement, flow cytometry, mixed genetic background analysis
Characterization of two distinct dendritic cell types in the injured cerebral cortex	Magnetic cell sorting, immune depletion of plasmacytoid dendritic cells, followed by qRT-PCR
Testing suppression of dendritic cells in the injured cortex by cyclophosphamide treatment	qRT-PCR, microarray expression analysis, flow cytometry

## Results

### *Ccl3* and *Ccr2* transcripts are differentially regulated in the injured cerebral cortex

**Figure 1:**
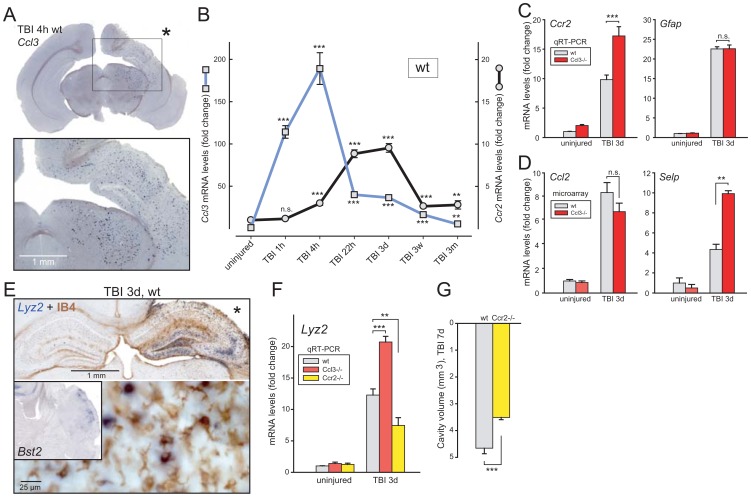
Traumatic brain injury (TBI) in wildtype (wt) and chemokine-deficient (*Ccl3*−/− and *Ccr2*−/−) mice. (**a**) *In situ* hybridization revealed intense *Ccl3* expression in the perilesional zone four hours after trauma, in a pattern resembling distribution of activated microglial cells. (**b**) Temporal patterns of *Ccl3* and *Ccr2* transcript levels in the injured wt neocortex revealed by qRT-PCR. (**c**) The *Ccr2* transcript was further upregulated in *Ccl3*−/− mice as compared to wt mice three days postinjury. *Gfap* expression was also strongly upregulated but with no differences between the strains. (**d**) The *Ccl2* transcript, encoding a Ccr2 ligand, was upregulated after injury with no differences between wt and *Ccl3*−/− mice as shown by microarray analysis. In contrast, *Selp* transcript expressed by endothelial cells was more upregulated in *Ccl3*−/− compared to wt brains. (**e**) *Lyz2* expression and the microglial surface marker isolectin B4 (IB4) showed only partial overlap three days post-injury in wt brains (*in situ* hybridization and histochemistry). The *Bst2*, characterizing pDCs, exhibited a different expression pattern (insert). (**f**) At three days postinjury, the *Lyz2* increase shifted in opposite directions in the *Ccl3*−/− and *Ccr2*−/− compared to wt brains. (**g**) Cavity volume was reduced in the *Ccr2*−/− compared to wt mice seven days after injury in accordance with downregulation of inflammatory *Lyz2*-response.

TBI rapidly results in local expression of chemokine *Ccl3* in the mouse neocortex [1]. By *in situ* hybridization, responding cells were seen regularly spaced in ipsilateral neocortex, hippocampus and subcortical structures including mesencephalon in a pattern indicating *Ccl3* expression in reactive resident microglia (Figure 1A). Quantitative RT-PCR (qRT-PCR) show increases in *Ccl3* transcript in neocortex one hour after injury with a peak after four hours (Figure 1B), expression remaining modestly increased one to three days. Also, the *Ccl2* transcript is upregulated within one hour after TBI [1]. The transcript of *Ccr2*, encoding a cognate receptor for Ccl2, became detectable four hours after injury with a peak at three days (Figure 1B). This temporal pattern may reflect delayed invasion of inflammatory cells from the periphery. Levels of both *Ccl3* and *Ccr2* transcripts remained well above background three weeks and three months after the injury.

To examine injury responses in the cerebral neocortex deficient in Ccl3 signaling, we studied TBI in *Ccl3*−/− mice [9]. Surprisingly, the *Ccr2* transcript was upregulated even further after three days in neocortex compared to wt mice (Figure 1C). In contrast, *Gfap* expression by reactive astrocytes was increased to the same extent in wt and *Ccl3*-knockout mice implying similar severity of the inflicted injury.

Considering the stronger increase of *Ccr2* transcripts in the injured cerebral cortex of *Ccl3* knockout mice, transcripts representing inflammatory functions were examined by unbiased microarray analysis. Comparison of wt and *Ccl3*−/− mice identified over 30 genes that fulfilled the following criteria: increased more than three-fold three days post-injury comparing injured with uninjured wt mice, upregulated at least 50% further in the injured *Ccl3*-deficient mice compared to wt mice. Identified transcripts involved in inflammatory responses included *C3ar1*, *Ccr2*, *Csf2rb1*, *Cybb*, *Dab2*, *H2-Aa*, *Il2rg*, *Msr2*, *Selp*, *Tgfbi*, *Thbs1* and *Tlr1*. In contrast to the increases in *Ccr2*, the cognate Ccr2 ligands *Ccl2* (Figure 1D, left) and *Ccl12*, possibly produced by reactive astrocytes, were equally enhanced in injured wt and *Ccl3*−/− brains as was *Gfap* expression. Thus, augmented ligand levels are not likely to account for the further increase of *Ccr2* in the injured *Ccl3*−/− mice. Rather, the strong increase in platelet selectin (*Selp*) in the injured *Ccl3*-deficient mice (Figure 1D, right) indicates endothelial involvement [1], [6] in recruitment of *Ccr2*-positive leukocytes attracted to the injured brain.

### *Ccr2* deficiency results in reduced *Lyz2* level and smaller cavity volume

We also examined the outcome of TBI in wt mice compared to homozygous *Ccr2*-deficient mice [10] three days postinjury. Lysozyme 2 (*Lyz2*) previously identified by microarray analysis as injury-induced in neocortex^1^ was among the transcripts found reduced in the injured *Ccr2−/−* cortex. From *in situ* hybridization, *Lyz2* expression was obvious in large phagocyte-like cells only partially overlapping with the activated microglia cell-surface marker isolectin B4 (IB4) [1] in injured neocortex and hippocampus (Figure 1E). This pattern contrasts with the clustered appearance of *Bst2* (bone marrow stromal cell antigen 2, encoding PDCA-1, plasmacytoid dendritic cell antigen 1) expressed by pDCs [11] (Figure 1E, insert left). qRT-PCR confirmed that *Lyz2* injury-increased expression was lower in *Ccr2*−/− compared to wt cortices. In contrast, *Lyz2* was further upregulated in injured *Ccl3*−/− brains compared to wt brains and thus in parallel with *Ccr2* expression levels (Figure 1F).

The injury-induced cortical cavity volume seen in wt brains was significantly reduced in the *Ccr2*−/− mice seven days after injury (Figure 1G). This is in line with the reduced upregulation of *Lyz2* in the injured *Ccr2*−/− cortex and suggests that fewer tissue-eliminating phagocytic cells invaded the injured brain when the Ccl2/Ccl12 attraction mediated by Ccr2 signaling was interrupted.

### Deletion of *Ccr2* does not affect injury-evoked *Ccl3* but reduces *Cxcl10* expression

**Figure 2:**
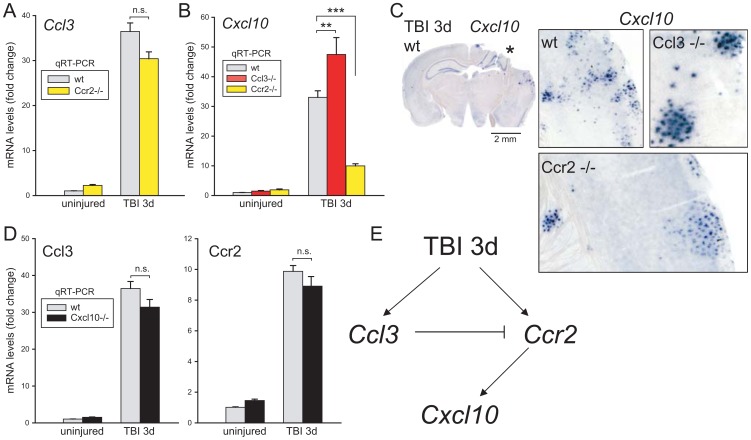
Interactions among chemokine transcripts analyzed with qRT-PCR and *in situ* hybridization in neocortex three days after TBI. (**a**) The *Ccl3* transcript expression did not differ between *Ccr2*−/− and wt brains. (**b**) Compared to wt mice, *Cxcl10* transcript showed opposite changes in expression in *Ccl3*−/− and *Ccr2*−/− brains. (**c**) A clustered cell pattern was seen for the *Cxcl10* transcript by *in situ* hybridization in all injured brains, although with different intensities depending on genotype. Expression was stronger in the injured *Ccl3*−/− mice and weaker in the *Ccr2*−/− mice compared to wt brains. (**d**) *Ccl3* and *Ccr2* upregulation showed no differences in wt compared to *Cxcl10*−/− injured brains by qRT-PCR. (**e**) Suggested model of interactions among chemokine ligands Ccl3 and Cxcl10 and the chemokine receptor Ccr2 three days after brain injury.

The *Ccl3* expression increased in concert in wt and *Ccr2*−/− mice (Figure 2A) whereas *Cxcl10* injury-induced expression [1], [2] was markedly dampened in brains lacking *Ccr2* (Figure 2B). In contrast, qRT-PCR showed that *Cxcl10* transcript was elevated above wt levels in the injured *Ccl3*-deficient brain, similar to shifts in *Ccr2* and *Selp* transcripts (Figures 1C and D). The increased *Cxcl10* expression appears in clustered inflammatory cells [1] and we earlier showed [2] that injured *Cxcl10*−/− mice [12] lacked this spotted staining. The clusters of positive cells appear with higher intensity in the injured *Ccl3*−/− cortex compared to wt (Figure 2C). In injured *Ccr2*−/− mice, expression of *Cxcl10* was evident in cell clusters but at reduced labeling intensity in line with the qRT-PCR data (Figure 2B).

*Cxcl10* expression did not affect injury-induced upregulation of *Ccl3* and *Ccr2* transcripts three days post-injury in traumatized *Cxcl10*−/− mice (Figure 2D). A model of chemokine interactions during the initial three days following injury is presented in Figure 2E. The injury resulted in independent increases in cortical levels of *Ccl3* and *Ccr2* transcripts. Since, *Ccr2* affected *Cxcl10* levels (Figure 2B), but not the reverse (Figure 2D), *Cxcl10* is positioned downstream and positively regulated by *Ccr2*. Furthermore, *Ccl3*-defiency increased the injury-evoked *Ccr2* levels (Figure 1C) indicating that *Ccl3* normally exerts a suppressive effect on *Ccr2* and thus indirectly limit *Cxcl10* expression in the injured brain.

### Number of dendritic cells is reduced in the injured *Ccr2*-deficient cortex

**Figure 3:**
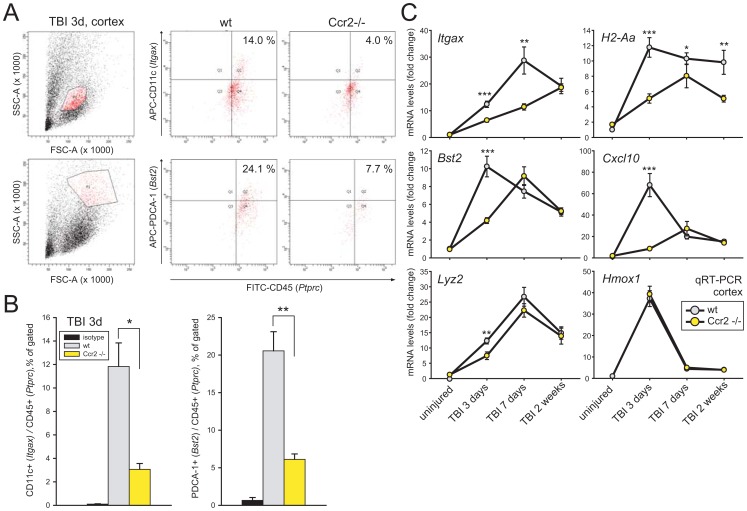
Inflammatory cells in the injured wt and *Ccr2*−/− cortex examined by flow cytometry and qRT-PCR. (**a**) Cells detected with FITC-CD45 in combination with either APC-PDCA-1 (*Bst2*) or APC-Cd11c (*Itgax*) in neocortex of wt or *Ccr2*−/− mice three days after injury. Upper panel left shows gating for Cd11c. The middle and right panels show wt and *Ccr2*−/− results, respectively, demonstrating reduced number of Cd11c-positive cells in the *Ccr2*−/− mice. Lower panel shows that PDCA-1 positive cells were reduced in *Ccr2*−/− brains. (**b**) Quantitative flow cytometry data from wt and *Ccr2*−/− mice three days postinjury. Isotype controls showed only trace signals. (**c**) Temporal expression patterns of six inflammatory-related transcripts (*Itgax*, *H2-Aa*, *Bst2*, *Cxcl10*, *Lyz2* and *Hmox1*) after injury in wt and *Ccr2*−/− brains.

We further investigated the role of Ccr2 signaling in appearance of inflammatory cells in the injured neocortex. Flow cytometry in fractionated dissociates from cortex three days after injury demonstrated the presence of both cDCs and pDCs in the injured neocortex (Figures 3A and B). Cd11c encoded by *Itgax* (alpha-X integrin) was used as a marker for cDCs [13]. pDCs [11] were characterized by PDCA-1 whereas CD45 (encoded by *Ptprc*) was used as a pan-leukocyte marker [1], [2]. Optimal gating for the two classes of dendritic cells (Figure 3A, left) identified double-positive cells (Figure 3A, right). Isotype controls gave low background signals whereas Cd11c- and PDCA1-positive cells increased less in the *Ccr2*-deficient compared to wt brains (Figure 3B). Uninjured brains showed only few cells double-positive for these markers. The data support the notion of impaired infiltration of peripheral inflammatory cells in mice lacking the Ccr2 chemokine receptor.

### Temporal shifts in inflammatory transcripts in the injured *Ccr2*−/− cortex

Unbiased microarray analysis identified genes less upregulated in *Ccr2*-deficient cortex compared to wt three days postinjury. Data revealed over 50 genes being upregulated more than two-fold in injured wt brains while reaching less than half of this level in *Ccr2*-deficient mice. In addition to *Cxcl10* and *Lyz2*, inflammatory transcripts included *Arg1*, *Chi3l3*, *Ccr1*, *Ccr5*, *Cybb*, *H2-Aa*, *Ifi204*, *Ifi205*, *Ms4a4c*, *Stat1*, *Tgfbi* and *Tlr1*. Microarray data were confirmed by qRT-PCR in a selection of these genes in *Ccr2−/−* mice and revealed persisting transcriptional differences one and two weeks postinjury (Figure 3C). Uninjured brains showed only trace expression of the examined transcripts. A reduction in injury-increases in the *Ccr2*−/− mice was seen for *Itgax* previously shown to be expressed in immune cells in the vicinity of *Cxcl10*-positive cell clusters [1], [2]. Cortical *Itgax* levels were reduced in the *Ccr2*−/− compared to wt brains and *Ccr2*−/− at three and seven days after injury. However, two weeks post-injury levels had increased equally in wt and mutant brains, indicating a delayed recruitment of antigen-presenting cDCs when *Ccr2* is missing. The *H2-Aa* transcript encoding an alpha chain in the antigen-presenting major histocompatibility complex class II (MHC II) was upregulated at three, seven days and two weeks postinjury in wt mice. The lower expression persisted in injured *Ccr2*−/− mice.

The *Bst2* transcript was distinctly upregulated in the injured wt cortex three days after injury but to a more modest degree in the *Ccr2*−/− mice (Figure 3C). At seven days after injury, levels of *Bst2* in the mutants reached the same levels as in wt mice. A slight reduction from these levels was seen two weeks after injury in both wt and *Ccr2*-deficient mice. The lack of a *Cxcl10* peak three days post-injury in the *Ccr2*−/− brains resembles the delayed expression of *Bst2*. However, at seven days postinjury the *Ccr2*−/− mutant had caught up with wt *Cxcl10* expression. The difference in *Lyz2* upregulation between wt and Ccr2−/− seen at three days did not persist at peak levels seven days after injury. In contrast, the heme oxygenase (decycling) 1 (*Hmox1*) transcript located juxta-positioned to the focal injury [1], was similarly upregulated in injured wt and mutant brains with a peak after three days (Figure 3C).

### Genetic background does not account for the differential effects of chemokine signaling

**Figure 4:**
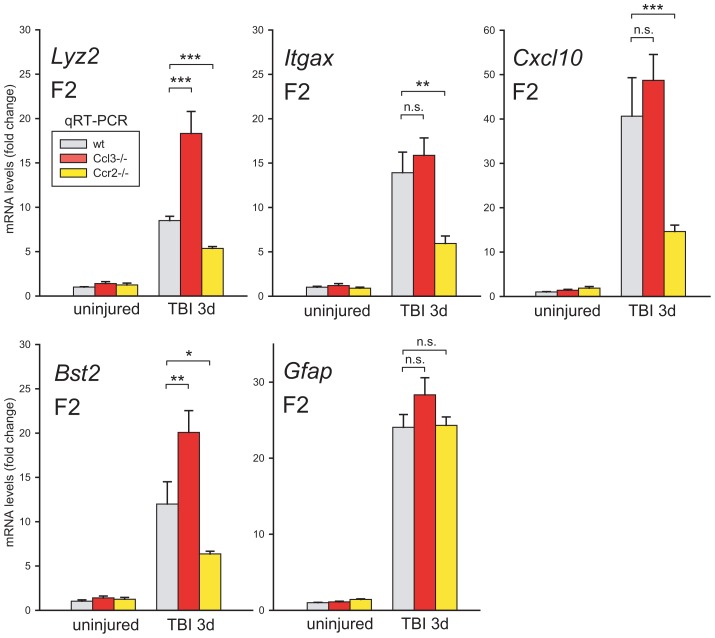
Inflammatory-related transcripts measured in *Ccl3*−/− and *Ccr2*−/− mice with F2 hybrid background three days postinjury. *Lyz2* expression was upregulated in homozygous *Ccl3* knockouts but downregulated in *Ccr2*−/− as in the parental strains, as was *Bst2*. Both *Itgax* and *Cxcl10* levels were less upregulated in the injured F2 Ccr2−/− brains. *Gfap* expression increased to the same extent in injured wt and F2 hybrid brains.

We tested for possible contribution from the genetic background to the strikingly opposite effects of *Ccl3-* and *Ccr2*-deficiencies (Figure 4). Crossing the inbred knockout strains resulted in expected Mendelian ratios of homozygous *Ccl3*−/− and *Ccr2*−/− mice in male F2 offspring, in line with location of these genes on chromosome 11 and 9, respectively. Cortical injury increased *Lyz2* levels in F2 *Ccl3*−/− mice whereas being reduced in F2 *Ccr2*−/− mice. The injury increases in *Itgax* and *Cxcl10* transcripts did not reach above wt levels in the F2 *Ccl3*−/− mice in contrast to *Bst2*. In the F2 *Ccr2*−/− cortices, *Lyz2*, *Itgax*, *Cxcl10* and *Bst2* were robustly reduced confirming results from the parental strains even in a mixed genetic background. It is of note that our genotyping of *Ccl3*-deficient mice does not distinguish *Ccr2*+/+;*Ccl3*−/− from *Ccr2*+/−;*Ccl3*−/− and this heterogeneity may contribute to the lack of upregulation of *Itgax* and *Cxcl10* in F2 *Ccl3*−/− mice. Irrespective of genotype and genetic background, *Gfap* levels were similar in all injured cortices.

### Antigen presentation marker is limited to classical dendritic cells

**Figure 5:**
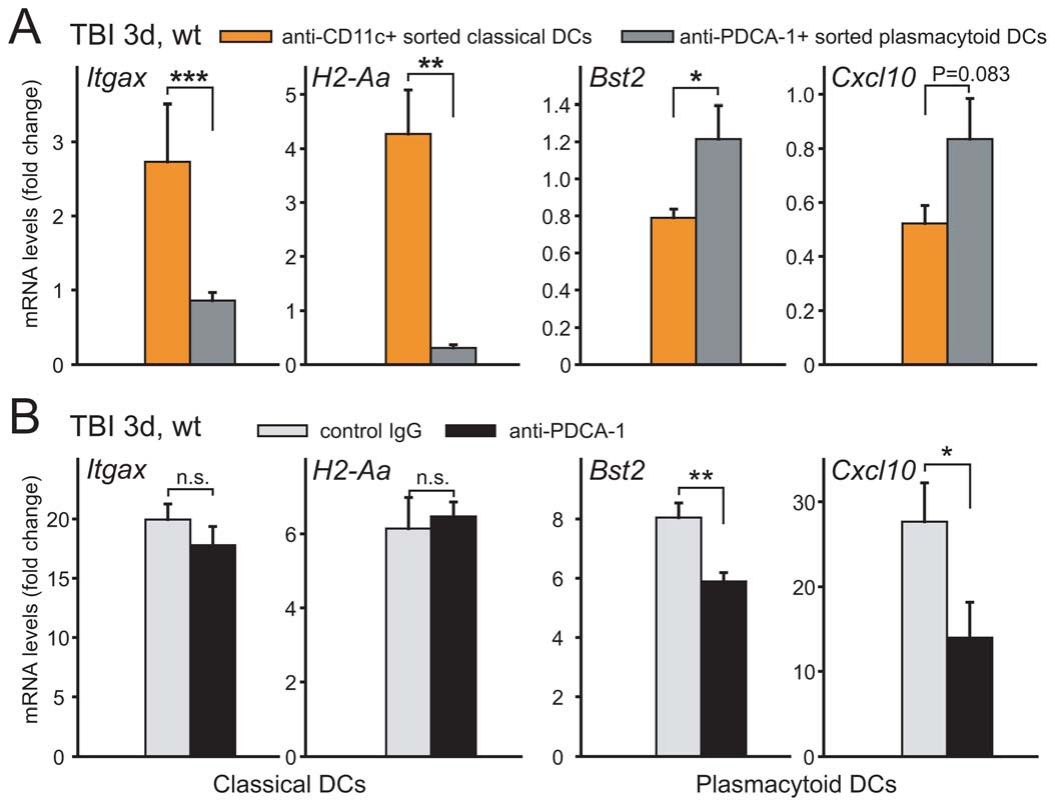
Cell sorting and immunodepletion of dendritic cells in neocortex three days after TBI in wt mice analyzed by qRT-PCR. (**a**) Cd11c-positive cells sorted on magnetic microbeads expressed enhanced *H2-Aa* and *Itgax* levels. In contrast, Bst2 was enriched in cells sorted on anti-PDCA-1 microbeads. (**b**) Cortical levels of inflammatory transcripts in mice injected with control immunoglobulin or with antibodies directed to PDCA-1. Depletion of pDCs was not accompanied by shifts in *Itgax* or *H2-Aa* transcript levels whereas a distinct reduction of *Bst2* and *Cxcl10* occurred.

Next, we isolated dendritic cell populations from wt neocortex three days after injury using anti-Cd11c and anti-PDCA-1 antibodies coupled to magnetic beads. Subsequent RNA analysis of the inflammatory cells sorted on anti-Cd11c showed enrichment of *Itgax* and *H2-Aa* transcripts (Figure 5A). Cells sorted on anti-PDCA-1 beads showed only weak expression of these two transcripts while highly expressing *Bst2*. In order to examine correlation between pDCs and *Cxcl10* expression, antibodies directed against PDCA-1 were administered to TBI mice and cells fractionated from the brain for RNA isolation. qRT-PCR showed that *Itgax* and *H2-Aa* transcripts were induced to the same extent in the injured neocortex whether the injected antibodies were directed against PDCA-1 or represented control IgG immunoglobulins (Figure 5B, left). In contrast, *Bst2* and *Cxcl10* transcripts were reduced in mice receiving anti-PDCA-1 antibodies compared to mice given equal doses of normal rat IgG (Figure 5B, right). These data support pDCs as the source of *Cxcl10* in injured neocortex.

### Cyclophosphamide treatment resembles Ccr2 deletion by limiting injury-induced transcripts

**Figure 6:**
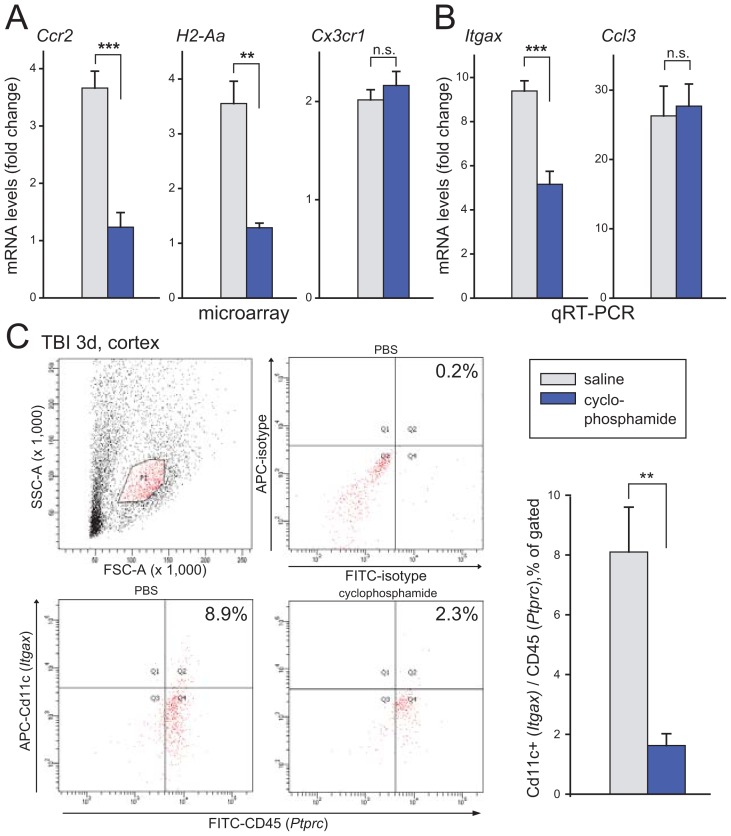
Cyclophosphamide treatment in wt mice analyzed three days after TBI. (**a**) Microarray showed markedly weakened injury-activated *Ccr2 and H2-Aa* levels while upregulation of the fractalkine receptor *Cx3cr1* was not affected. (**b**) *Itgax* was also dampened in mice subjected to cyclophosphamide treatment as shown by qRT-PCR whereas *Ccl3* upregulation was not impaired. (**c**) Flow cytometry demonstrated reduced number of brain cells gated for Cd11c in mice treated with cyclophosphamide. Isotype controls (upper middle panel) showed only trace labeling of inflammatory cells. Quantitative data from flow cytometry assays (right), show a substantial reduction of Cd11c-positive cDCs in the injured brains treated with cyclophosphamide compared to mice injected with saline.

We finally turned to intraperitoneal administration of the chemotherapeutic and anti-inflammatory agent cyclophosphamide to examine possible effects on injury-induced inflammatory cells in wt cerebral cortex three days postinjury. Microarray data from the cyclophosphamide-treated mice were compared to corresponding data from injured Ccr2−/− and wt mouse neocortex. Analysis of these three groups identified 20 genes that fulfilled the criteria of being upregulated more than two-fold in injured wt neocortex and reaching levels less than half of this in both *Ccr2*−/− mice and cyclophosphamide-treated mice (Table 3). Several of these transcripts are involved in inflammatory processes e.g. *Ccr2*, *Chi3l3*, *H2-Aa*, *Ifi204*, *Ms4a4c*, *Plac8*, *Stat1* and *Tgfbi*. The *Ccr2* reduction by cyclophosphamide in concert with transcripts supporting antigen presentation indicates a major effect on cDCs (*H2-Aa, Itgax*; Figures 6A and B). *Lyz2* and *Cxcl10* transcripts (qRT-PCR; n.s.) were not affected whereas a slight reduction of *Bst2* (qRT-PCR; P<0.05) suggests marginal influence of cyclophosphamide on pDCs but not phagocytes/macrophages. The upregulation of *Cx3cr1* encoding the fraktalkine receptor and *Ccl3* (Figures 6A and B), both characteristic of resident microglia, as well as *Gfap* and *Hmox1* (qRT-PCR; n.s.), were not affected by the cyclophosphamide treatment.

**Table 3:** Transcripts increased at least two-fold three days postinjury in wt cerebral cortex and with upregulation reduced by at least 50% in *Ccr2*−/− mice as well as in wt mice treated with cyclophosphamide.

Gene Symbol	Gene Title	GenBank	Probe Set ID
***Apoc2***	apolipoprotein C-II	NM_009695	1418069_at
***Ccr2***	chemokine (C-C motif) receptor 2	NM_009915	1421186_at
***Chi3l3***	chitinase 3-like 3	NM_009892	1419764_at
***Ddx60***	DEAD (Asp-Glu-Ala-Asp) box polypeptide 60	NM_001081215	1451777_at
***H2-Aa***	histocompatibility 2, class II antigen A, alpha	NM_010378	1435290_x_at
***Ifi27l2a***	interferon, alpha-inducible protein 27 like 2A	NM_029803	1426278_at
***Ifi35***	interferon-induced protein 35	NM_027320	1445897_s_at
***Ifi44***	interferon-induced protein 44	NM_133871	1423555_a_at
***Ifi203***	interferon activated gene 203	NM_001045481	1452231_x_at
***Ifi204***	interferon activated gene 204	NM_008329	1419603_at
***Ifi205***	interferon activated gene 205	NM_172648	1452349_x_at
***Isg15***	ISG15 ubiquitin-like modifier	NM_015783	1431591_s_at
***Ms4a4c***	membrane-spanning 4-domains, subfamily A, member 4C	NM_022429	1450291_s_at
***Mx1***	myxovirus (influenza virus) resistance 1	NM_010846	1451905_a_at
***Plac8***	placenta-specific 8	NM_139198	1451335_at
***Pyhin1***	pyrin and HIN domain family, member 1	NM_175026	1435331_at
***Sirpb1a***	signal-regulatory protein beta 1A	NM_001002898	1448025_at
***Stat1***	signal transducer and activator of transcription 1	NM_009283	1450034_at
***Tgfbi***	transforming growth factor, beta induced	NM_009369	1415871_at
***Usp18***	ubiquitin specific peptidase 18	NM_011909	1418191_at

Flow cytometry three days after injury treated with cyclophosphamide showed a robust drop in number of inflammatory CD45 (*Ptprc*) and Cd11c (*Itgax*) double-positive cells isolated from neocortex (Figure 6C) supporting RNA data at the protein/cell levels. Double isotype labeling in the area gated for cDCs yielded low background. The reduced Cd11c expression after cyclophosphamide treatment was confirmed by repeated experiments (Figure 6C, bottom right). Overall, these results demonstrate that it is feasible to interact pharmacologically with selected inflammatory cells after brain trauma as summarized in Table 4.

**Table 4:** Responding immune cells/cells responding in TBI.

Cell type	Expressed markers and molecules
Activated microglia	*Ccl3, Cx3cr1*, IB4-binding
Phagocytes/macrophages	*Ccr2, Lyz2*
Plasmacytoid dendritic cells	*Ccr2*, *Bst2*/PDCA-1, *Cxcl10*, CD45
Classical dendritic cells	*Ccr2, Itgax*/Cd11c, *H2-Aa*, CD45

## Discussion

The present findings confirm that interactions among chemokines in the injured brain set the stage for inflammatory cell activation. While resident microglia and astrocytes become engaged, a flow of invading immune cells from the periphery after trauma is also evident.

In particular, the current data demonstrate a strong activation of dendritic cells in the injured brain depending on immigration of *Ccr2* positive cells from the peripheral circulation [14]. Our data support a pivotal role of infiltrating cells of monocyte lineage in disease progression as demonstrated in previous reports using parabiotic mice and genetic deletion of the *Ccr2* receptor [15]. We show that this diapedesis is valid for cDCs [4], pDCs [16] and large phagocytes expressing *Lyz2*. The activation of dendritic cells in the injured cortex is distinct from the inflammatory response among resident, locally renewing microglia [17]–[19] characterized by *Cx3cr1* expression [20] and found to exert important surveillance functions in the brain [3].

cDCs express integrin alpha X (*Itgax*/Cd11c) and major histocompatibility class II complex (MHC II, e.g. *H2-Aa*) [21]–[23]. These cells coordinate a host of immune responses by capturing and processing proteins to peptides that are presented to T cells on MHC II [4]. Our data link *H2-Aa* with *Itgax*-expressing DCs serving as professional antigen presenting cells. These cells have been demonstrated to sufficiently trigger auto-reactive encephalitogenic T cells and to initiate CNS inflammation [24]. A distinct focal co-localization of cDCs and T cells in the inflamed brain, highly reminiscent of the current injury-induced inflammatory cell clusters, was shown and suggested to be indicative of CNS acting as a neolymphoid tissue [24]. The present results also connect pDCs, characterized by the *Bst2* transcript [11], [25] and its encoded protein PDCA-1, with the expression of *Cxcl10* in injured mouse cortex, in agreement with reports on CXCL10 production in activated human pDCs [26], [27].

Injury-induced activation of the chemokine Ccl2/Ccr2 axis is demonstrated by the current data. Previously, monocytes have been identified to express *Ccr2* as well as high levels of Ly-6C and found to be crucial for autoimmune inflammation in the CNS [28], offering a target for treatment strategies [29]. An important role for the *Ccr2* receptor in *Ccl2* attraction of monocytes has been demonstrated [10]. Moreover, using a selective Ccr2 antagonist reduced apoptosis while improving behavioral performance in a rat TBI model [30]. Currently, a drastic increase in *Ccr2*-linked transcripts was observed in the injured *Ccl3*-deficient brains. This is not attributable to increased Ccl2 levels attracting inflammatory *Ccr2* positive cells. A plausible explanation is offered by enhanced upregulation of platelet selectin, encoded by *Selp*, found in the vasculature of the injured *Ccl3*−/− brains. The *Ccl3*−/− mice have been shown to exhibit normal numbers of hematopoietic progenitor cells in peripheral blood and bone marrow and of T cells in lymph nodes and spleen [9]. The pathway by which Ccl3 is regulating *Selp* expression remains unknown but may result in curtailed endothelial expression of *Selp* and position *Ccl3* as a suppressor of *Ccr2*-expressing cells in the injured neocortex.

The present data also demonstrate that large phagocytic cells expressing *Lyz2* depend on invading *Ccr2*-positive cells and do not overlap with activated endogenous microglial cells. The injury-induced cortical cavity formation was reduced in our *Ccr2*−/− mice after seven days. This is in parallel with the reduction of *Lyz2* and adds to the idea that *Ccr2* is a key receptor in recruitment of different invading peripheral cells. A knock-in strategy to express Cre recombinase from the *Lyz2* locus in mice [31] showed high expression of *Lyz2* in macrophages and only low expression in Cd11c+ splenic dendritic cells. Also, absence of Ccr2 reduced infarct sizes in a mouse model of cerebral ischemia/reperfusion injury [32], correlating with the current demonstration of reduced cortical cavity after TBI in *Ccr2*-deficient mice.

The alkylating agent cyclophosphamide affects DNA synthesis e.g. in immune cells in mice [33], acting as an immunosuppressant with clinical applications in autoimmune diseases such as systemic lupus erythematosus and rheumatoid arthritis. Presently, an impaired invasion of inflammatory cells in injured wt brain receiving cyclophosphamide administration was shown by restriction of injury-evoked *Ccr2*, *Itgax* and *H2-Aa* expression linked to a reduction of inflammatory Cd11c/*Itgax* positive cDCs. In contrast, cyclophosphamide did not affect the injury-elicited upregulation of *Ccl3* or *Cx3cr1* in resident microglia, had only marginal effects on pDCs and showed no manifest influence on phagocytes.

The current inflammatory response in the injured brain does not obviously fit the concept of Th1/M1 versus Th2/M2 divergence in macrophage functions [34]. Our microarray data show injury-evoked changes in transcripts assigned to pro-inflammatory, classically activated M1 (characterized by e.g. *Cxcl10*, *Il6* and *Il1*) and anti-inflammatory, alternatively activated M2 macrophages (associated with *Arg1*, *Chi3l3*, *Il4ra*, *Il13ra1*, *Tgfb1*, *Msr1* and *Cebpd*) [35], [36]. Among Th2/M2-transcripts, *Chi3l3* was less upregulated both in injured *Ccr2*−/− brains and cyclophosphamide-treated wt brains. Thus, the present injury model engaged transcripts in both of these pathways.

To what extent do the current findings from the injured mouse brain apply to human clinical conditions? A poor resemblance of inflammatory responses in humans and mice has been suggested from large scale genomic studies of systemic inflammation [37]. One particular case is the *Selp* promoter that is strongly upregulated in the mouse by various insults but unresponsive to inflammation in humans [38]. However, *Selp* may have other functional counterparts in the endothelia of patients. Nevertheless, in general the present findings are in line with observations of both cDCs and pDCs in patients with neurological conditions involving inflammation [39]. Also, several of the currently identified inflammatory transcripts are associated with human injuries. Thus, *CCL2* and *CCL3* transcripts were both increased in patients with posttraumatic brain contusion [40]. CXCL10, CCL3 and CCL2 were upregulated in plasma and microdialysis perfusates in TBI patients [41], [42] and CCL2 found elevated in cerebrospinal fluid [43]. Moreover, increased levels of the CCL2 protein in serum of both civilian TBI patients and military blast-induced mild TBI cases have been demonstrated and found to be a potential risk factor for subsequent dementia [44]. Finally, the human orthologue of mouse *H2-Aa*, known as *HLA-DQA1*, encodes one of the HLA class II alpha chains and has been associated with autoimmune conditions including asthma, myasthenia gravis and celiac disease [45]–[47]. Taken together, our results suggest similarities in the studied systems between man and mouse.

The present data demonstrate separate repertoires among invading inflammatory cells of monocytic lineage distinct from those of activated resident cells. Moreover, a potential of dampening specific inflammatory cells invading the injured brain by pharmacological means is revealed. Thus, our findings suggest a time-window for therapeutic interference of invading *Ccr2*-positive, antigen-presenting cells after traumatic brain injury.

## Materials and Methods

### Traumatic brain injury (TBI)

Male C57BL/6 (B6) mice with a body weight of 25–35 g were used for the traumatic brain injury (TBI). The mice were anaesthetized with 3.5% isoflurane (in combination of 70% nitrous oxide and 30% oxygen) before being transferred to a stereotactic frame. Anaesthesia was continued with 1–2% isoflurane, using mask ventilation and spontaneous breathing for the rest of the procedure. Bupivacaine (0.5 mg) was injected subcutaneously in the neck providing local analgesia. Body temperature (37°C) was controlled rectally throughout surgery. A craniotomy (approximately 3 mm diameter) was made over the right parietal cortex between midline, bregma and lambda. The mice were subjected to controlled cortical impact (CCI) injury [7], [8] by a pneumatic impact device (model AMS 201, AmScien Instruments, Richmond, Virginia, USA). The compression depth was 0.5 mm, compression duration 100 ms and the velocity 3.1 m/s, resulting in a severe, focal injury. Control tissues were from uninjured mice without craniotomy. All efforts were made to minimize animal suffering in compliance with ARRIVE (Animal Research: Reporting of *In Vivo* Experiments) guidelines. Uppsala Ethical Committee on Animal Experiments evaluated and authorized all experimental protocols.

### In situ hybridization

Mice were deeply anaesthetized and perfused by a cardiac infusion of sodium chloride followed by 4% paraformaldehyde. Brains were postfixed in formaldehyde overnight. A vibratome was used to cut 60 µm coronal slices collected in phosphate-buffered saline, dehydrated in methanol and stored at −20°C over night. Sections were rehydrated in methanol and saline containing 0.1% Tween-20, bleached in 6% H_2_O_2_, treated with 0.5% Triton X-100 and permeabilized with proteinase K and postfixed in formaldehyde. Antisense riboprobes encoding *Ccl3*, *Cxcl10*, *Lyz2* and *Bst2* were synthesized from linearized IMAGE clones using SP6, T3 or T7 polymerase and DIG RNA Labeling Kit (Roche, Mannheim, Germany). Analysis was based on several sections respresenting at least two injured brains subjected to repeated hybridizations giving similar results. Sections were prehybridized two hours at 55°C. Probes (1 µg/ml) were denatured at 80°C and added to the sections for incubation at 55°C over night. Anti-DIG antibody was diluted 1∶5,000 in blocking solution for an overnight incubation at 4°C. Levimasole was used to inhibit endogenous phosphatase. Finally, sections were developed with BM-purple alkaline phosphatase substrate (Roche) at 37°C before being washed in saline, mounted onto microscope slides and photographed.

### RNA preparation

Neocortex from the injured side of the brain was dissected and stored in the RNA*later* reagent (Qiagen Inc., Valencia, California, USA). The tissue was homogenized using a Polytron homogenizer and total RNA isolated by RNeasy Mini kit (Qiagen) with absorbance determined at 260 and 280 nm. RNA was prepared from uninjured wt mice (n = 10) and at the following time points after injury: one hour (n = 3), four hours (n = 4), twenty-two hours (n = 4), three days (n = 13), seven days (n = 11), two weeks (n = 12), three weeks (n = 9) and three months (n = 9). Moreover, the *Cxcl10*−/− RNA consisted of uninjured mice (n = 2) and injured mice at three days (n = 7) and seven days (n = 9) postinjury. RNA from *Ccl3*−/− brains were represented by uninjured mice (n = 2) and injured at three days (n = 9) and seven days postinjury (n = 5). The *Ccr2*−/− RNA comprised of two uninjured brains and postinjury at three days (n = 9), seven days (n = 11), two weeks (n = 11) and three weeks (n = 6). The RNA from the injured F2 hybrids consisted of five brain samples of *Ccl3*−/− mice and six samples of *Ccr2*−/− mice that were compared with five injured wt mice.

### Quantitative reverse-transcriptase PCR (qRT-PCR)

Total RNA (10 ng) was analyzed and measurements were repeated at least twice using duplicated microwells (25 µl reaction volume). Injury-induced transcriptional changes were studied using the following primer pairs (reference sequence number and upper as well as lower primer stated): *Ccl3* (NM_011337, 5′-GCC TGC TGC TTC TCC TAC AG-3′ and 5′-TCT GCC GGT TTC TCT TAG TC-3′), *Ccr2* (NM_009915, 5′-TGC CAT CAT GAT TGT CTA CTT T-3′ and 5′-CCT GCA TGG CCT GGT CTA AGT-3′), *Cxcl10* (NM_021274, 5′-ACC CAA GTG CTG CCG TCA TT-3′ and 5′-ATT CTC ACT GGC CCG TCA TC-3′), *Gfap* (NM_010277, 5′-CGG GAG TCG GCC AGT TAC CAG-3′ and 5′-TTT CCT GTA GGT GGC GAT CTC-3′), *Lyz2* (NM_017372, 5′-ATG GGT GGC ATG GCG AGC AC-3′ and 5′-TGA GAA AGA GAC CGA ATG AG-3′), *Itgax* (NM_021334, 5′-ACA CAG TGT GCT CCA GTA TGA-3′ and 5′-GCC CAG GGA TAT GTT CAC AGC-3′), *Bst2* (NM_198095, 5′-GCG CCC TCT TTC TAT CAC TA-3′ and 5′-GAC AAT CTA CTT CGC CGT CA-3′), *H2-Aa* (NM_010378, 5′-CAA CTT GGG AGT CTT GAC TA-3′ and 5′-CAG GAG GGA AGA TGT TGT C-3′) and *Hmox1* (NM_010442, 5′-ACC TTC CCG AAC ATC GAC AG-3′ and 5′-GAG CAG GAA GGC GGT CTT AG-3′). The iScript One-Step RT-PCR Kit with SYBR Green (Bio-Rad Laboratories, Inc., Hercules, California, USA) was used. Reverse transcription was run for 10 min at 50°C. Thereafter, qRT-PCR was initiated by a hot-start at 95°C during 5 min followed by 36 cycles (95°C for 10 s, 60°C for 30 s) using a CFX96 thermal cycler (Bio-Rad). Melting curves were obtained by temperature increments of 0.5°C from 55.0°C to 94.5°C. Expression of 28S rRNA (X00525, 5′-GGG AGA GGG TGT AAA TCT CGC-3′ and 5′-CTG TTC ACC TTG GAG ACC TGC-3′) was used as a reference. Threshold cycle differences (ΔΔCt) were transformed to linear fold changes with reference to equal amounts of total RNA in uninjured cortex.

### Genetically modified mice

TBI was performed in three strains of chemokine signaling deficient mice. Ccl3 knockout B6.129P2-*Ccl3^tm1Unc^*/J (JAX #002687) [9] and Ccr2 knockout B6.129S4-*Ccr2^tm1Ifc^*/J (JAX #004999) [10] were obtained as homozygous females crossed with our local B6 mice. Cxcl10 knockout B6.129S4-*Cxcl10^tm1Adl^*/J (JAX strain #006087 Jackson Laboratories, Bar Harbor, Maine, USA) [12] was obtained as homozygous breeding pairs as described previously [2]. The strains were then maintained by crossing heterozygotes or homozygotes to produce the required number of homozygous males for TBI. All experiments used knockout mice backcrossed for at least nine generations with B6 wt mice. The three chemokine-defect strains of mice exhibit no overt phenotypic deficiencies, impaired development or fertility, nor did the targeted alleles deviate from expected Mendelian ratios. Control wt B6 males were from our local breeding colony or obtained commercially. In order to test for any contribution from the genetic background of the knockout mice, hybrid F1 *Ccl3*+/−;*Ccr2*+/− mice were created to serve as parents for the F2 hybrid generation. For genotyping, tail-tips from three-week-old pups were taken and the pups marked by ear tags. The tail-tips were put in 0.3 ml lysis buffer (0.5 ml 0.1 M Tris-HCl buffer pH 8.5 with 5 mM EDTA, 0.2% SDS and 0.2 M NaCl with proteinase K freshly added to 0.1 mg/ml final concentration) and incubated overnight at 60°C. For DNA-precipitation, equal volume of isopropanol was added. Genotyping by PCR was performed with the primers specified below using AmpliTaq Gold polymerase (Applied Biosystems, Foster City, California, USA) using the following steps: 35 cycles of 94°C 30 s, 56°C 45 s and 72°C 60 s. Primers for *Cxcl10* (NM_021274, 5′-ACC CAA GTG CTG CCG TCA TT-3′ and 5′-ATT CTC ACT GGC CCG TCA TC-3′) and yielded a 650 base-pair fragment. The *Ccr2* primers (NM_009915, 5′-TCC TTG GGA ATG AGT AAC TG-3′ and 5′-TTA CAA CCC AAC CGA GAC-3′, respectively) resulted in a band size of 274 base pairs. The *Ccl3* primers (NM_011337, 5′-GCC TGC TGC TTC TCC TAC AG-3′ and 5′-TCT GCC GGT TTC TCT TAG TC-3′) gave a fragment length of 350 base pairs. In addition, to detect the neomycin resistance cassette we used primers (U43612, 5′-CTT GGG TGG AGA GGC TAT TC-3′ and 5′-AGG TGA GAT GAC AGG AGA TC-3′) yielding an amplicon length of 280 base pairs. Fragment size was evaluated by gel electrophoresis (using 1.4–2.0% agarose gels with 0.005% ethidium bromide) followed by examination by UV light in a ChemiDoc imager (Bio-Rad, Hercules, California, USA).

### Microarray analysis

We used unbiased microarray technique with samples from the mouse neocortex hybridized to GeneChip® Mouse Genome 430 2.0 Arrays (Affymetrix, Inc., Santa Clara, CA, USA). The material included arrays from uninjured wt (n = 10), *Ccl3*−/− (n = 2) and *Ccr2*−/− (n = 2) mice. In addition, arrays included wt three days postinjury (n = 12) as well as *Ccl3*−/−, *Ccr2*−/− and wt/cyclophosphamide three days (n = 5 respectively). Sample analysis was carried out at The Uppsala University Hospital Array Platform using Affymetrix Microarray Suite version 5.1 (MAS 5.1) applying the percentile algorithm (Parameters = Percentile:75, MAS5-Detection P = 0.05). The analysis excluded data that were unassigned ESTs, probes with signal intensities below 38 and also eliminating signals indicated as Absent. Microarray data can be retrieved in the National Center for Biotechnology Information (NCBI) Gene Expression Omnibus (GEO) database (http://www.ncbi.nlm.nih.gov/geo/query/acc.cgi?acc=GSE58485), accession number GSE58485.

### Flow cytometry

The neocortex of uninjured and TBI mice (three days postinjury) were dissected (n = 1–2, wt or *Ccr2−/−* mice, experiments in triplicate) and treated with collagenase D. Cells from the brain tissue were mechanically isolated using a loose fitted (0.1 mm) Dounce homogenizer in Hank's balanced salt solution (containing phenol red) with 50% Percoll (GE Healthcare, Uppsala, Sweden) and DNAse I. The cells were fractionated by centrifugation using a discontinuous Percoll gradient. Cells collected at the 63%/50% and 50%/30% Percoll interphases were pooled. To reduce unspecific staining, the Fc receptors of the cells were blocked with anti-mouse CD16/CD32 (Fc block from BD Biosciences, San Jose, CA, USA), and subsequently stained with fluorescein isothiocyanate (FITC)-labeled CD45.2 antibodies (BD 553772) together with either allophycocyanin (APC)-labeled CD11c (*Itgax*, BD 550261) or AlexaFluor647-labeled PDCA-1 (*Bst2*, eBio 12-5931-82). To determine background, FITC-labeled isotype mouse IgG2aĸ (BD 553456) was combined with APC-labeled isotype of either hamster IgG1 (BD 553956) or rat IgG2b (BD 553991). The cell suspensions were then passed through a cell strainer with 40 µm meshes and analyzed in a FACS CANTO II flow cytometer (BD Biosciences).

### Histochemistry

To detect activated microglia, peroxidase-labeled isolectin B4 from *Bandeiraea simplicifolia* (L5391, Sigma, Saint Louis, Missouri, USA) was applied to sections, previously hybridized with the *Lyz2* probe. Binding of isolectin B4 was examined in duplicate sections by adding DAB as a peroxidase substrate before being mounted.

### Injury-induced cavity volume analysis

Mice were perfused by 4% paraformaldehyde one week after injury. The brains were removed, cryoprotected in 30% sucrose, snap-frozen in ice-cold isopentane and stored at −80°C. Brains were embedded in Tissue-Tek (Histolab, Gothenburg, Sweden) and cut in 20 µm coronal sections with a cryostat, beginning at bregma. Every 25th section was saved on glass slides (SuperFrost Plus, Menzel-Gläser, Braunschweig, Germany). After nuclear staining in Harris hematoxylin (Histolab), slides were dehydrated and mounted. Images of sections of the injured brains were stored in a digitized format for area measurements in order to reconstruct the cavity volume. Ipsilateral area was subtracted from the corresponding contralateral and area difference was transformed into a circular area to calculate the radius. The total cavity volume was approximated as the sum of the volume of each truncated cone, stacked in the injury. Measurements were performed in six *Ccr2*−/− mice and five wt mice.

### Magnetic cell sorting

Inflammatory cells fractionated on Percoll gradients, were incubated with magnetic microbeads coated with anti-Cd11c or anti-PDCA-1 antibodies (Miltenyi Biotech, Bergisch Gladbach, Germany). Four independent experiments were performed, each analysis based on cells pooled from neocortex from two up to six injured mice. The magnetic cell sorting was accomplished using MS Columns and a MiniMACS separation unit (Miltenyi Biotech) for subsequent recovery of RNA. RNA was analyzed in duplicate with qRT-PCR using *Itgax*, *H2-Aa*, *Bst2* and *Cxcl10* probes and normalized against 28S ribosomal RNA.

### Depletion of plasmacytoid dendritic cells

Wt mice (n = 6) were injected intraperitoneally 30 minutes after injury with 500 µg rat antibodies directed against mouse PDCA-1 (# 130-091-978, Miltenyi Biotech, Bergisch Gladbach, Germany) to achieve depletion of pDCs. As reference, the same amount of normal rat IgG (I4131, Sigma Aldrich Sweden AB) were administered to wt mice (n = 8). Brains were collected for RNA preparation three days after injury.

### Cyclophosphamide treatment

Wt mice were given phosphate buffered saline (n = 6) or cyclophosphamide monohydrate (n = 8), 200 mg/kg; C0768, Sigma Aldrich) intraperitoneally 30 minutes after the TBI and tissue collected three days later for RNA preparation for qRT-PCR and microarray analysis. In addition, injured mice given saline (PBS) or cyclophosphamide were analyzed by flow cytometry.

### Statistical analysis

The SigmaStat version 3.1 software (SPSS, Inc., Richmond, CA, USA) was used to perform One Way Analysis of Variance (ANOVA) or Student's *t*-test for pair-wise comparisons of groups. Non-parametric data were analyzed using Kruskal-Wallis One Way Analysis of Variance on Ranks or Mann-Whitney *U* test as advised by the program. P = 0.05 or lower was considered to represent statistically significant differences. Data in the text and graphs are presented as mean values ± standard error of the mean (SEM).
